# The Effect of Maternal Adverse Childhood Experiences (ACEs) on Substance Use During Pregnancy

**DOI:** 10.1007/s10995-023-03768-4

**Published:** 2023-09-21

**Authors:** Shae Duka, Sadeea Rahman, Susan E. Hansen, Debra Esernio-Jenssen

**Affiliations:** 1 Network Office of Research and Innovation, Lehigh Valley Health Network, Allentown, USA; 2 Morsani College of Medicine, University of South Florida, Tampa, USA; 3 Department of Pediatrics, Jacobi Medical Center, New York, USA; 4 Department of Pediatrics, Lehigh Valley Health Network, Allentown, USA; 5 Lehigh Valley Reilly Children’s Hospital, Cedar Crest Boulevard and I-78, Allentown, PA 18103 USA

**Keywords:** Adverse childhood experiences, Substance use in pregnancy, Neonatal drug exposure

## Abstract

**Objectives:**

To analyze adverse childhood experiences (ACEs) among mothers of newborns referred to a hospital’s child protection team (CPT) for suspected substance exposure. Researchers hypothesized that a higher prevalence of these mothers have ≥ 4 ACEs than female counterparts in the general population. The study team also explored whether associations existed between type of maternal ACEs and substance use in pregnancy.

**Methods:**

Retrospective review of infant referrals to the CPT in the 3 years after adding an ACEs questionnaire to the consultation process. Bivariate analyses and multivariate logistic regression models examined associations between prenatal substance use and maternal ACEs prevalence, controlling for demographics.

**Results:**

Data from 222 infants (four sets of twins) and 218 mothers were analyzed. Half (50.0%) the infants had withdrawal symptoms. Most (67.0%) women had positive toxicology screens, while 85.0% reported prenatal substance use. Half (50.9%) the mothers reported ≥ 4 ACEs and these individuals had significantly higher odds of cannabinoid use [adjusted odds ratio (aOR), 3.7; 95%CI 2.0, 6.9, p < 0.001) than those with < 4 ACEs. A significant association was found between substance use and ACEs in the household challenges category (p = 0.03), especially parental separation/divorce (p < 0.001).

**Conclusions for Practice:**

As hypothesized, a higher prevalence of mothers referred to the CPT had ≥ 4 ACEs than women in the general population (50.9% vs. 15.2%), and a large proportion had used substances while pregnant. Routine prenatal ACEs screening and universal, nonpunitive toxicology testing of infants and mothers at birth may provide opportunities for intervention while reducing the transgenerational impact of ACEs.

## Significance

It is known that Adverse Childhood Experiences (ACEs) put people at risk of poor health choices and outcomes later in life. Pregnant people who have experienced ACEs—especially those with ≥ 4 events —are at risk for prenatal substance use, exposing their infants in utero. The high prevalence of people in this study with ≥ 4 ACEs whose infants tested positive for substance exposure provides further evidence of the association between ACEs and substance use in pregnancy. Prenatal screening for childhood adversity may identify people at risk and provide opportunities for intervention, thus reducing the transgenerational impact of ACEs.

## Introduction

Adverse childhood experiences (ACEs) have been shown to affect health throughout a person’s lifetime and the more events experienced increases one’s risk of poor health outcomes in what is referred to as a dose–response relationship (Felitti et al., 1998; Shonkoff et al., 2012; Smith et al., 2016). Parents with multiple ACEs are at highest risk for health behaviors that create ACEs for their children (e.g., substance use, violence, and mental illness) (Centers for Disease Control and Prevention (CDC), 2016; Duffy et al., 2018; Felitti et al., 1998; Hughes et al., 2017; Knight et al., 2014; Merrick et al., 2018).

According to data reported by the CDC from the original ACEs study (CDC, 2016; Felitti et al., 1998) conducted in California (n = 17,337), 15.2% of women and 9.2% of men had experienced four or more (≥ 4) ACEs before age 18. A more recent report, which included data from 23 states (n = 214,157), shows an even higher prevalence of those with ≥ 4 ACEs (17.8% of women, 13.7% of men) (Merrick et al., 2018).

ACEs scores are frequently reported as a composite, or cumulative, score generated from the positive responses to a list of contextual experiences or traumatic events occurring in the first 18 years of life (Felitti et al., 1998). They also have been examined categorically (e.g., abuse, neglect, household dysfunction/challenges) (Felitti et al., 1998; Ford et al., 2014) to determine whether specific types of ACEs have greater impact on health outcomes. For example, the original ACEs study (Felitti et al., 1998) found that women most frequently (29.5%) reported substance use by someone in their household (ACEs category “household dysfunction”) followed by 27.0% who reported physical abuse (ACEs “abuse” category) (CDC, 2016), while the expanded study data show emotional abuse (33.9%, abuse category) as the most prevalent, followed by substance use in the household (28.7%, household dysfunction category) (Merrick et al., 2018).

### ACEs and Pregnancy Outcomes

Women with ACEs score ≥ 4 have been shown to be at greater risk of poor outcomes in pregnancy (Christiaens et al., 2015; Lê-Scherba et al., 2018). One of the long-term outcomes with which ACEs have been associated is substance use in adulthood (Scheidell et al., 2018), including during pregnancy (Chung et al., 2010; Currie & Tough, 2021; Frankenberger et al., 2015; Leeners et al., 2014; Nguyen et al., 2019; Olsen, 2018; Racine et al., 2020). Women with higher ACEs scores have been shown to be two to six times more likely to use tobacco (Chung et al., 2010; Racine et al., 2020), alcohol (Chung et al., 2010; Frankenberger et al., 2015; Racine et al., 2020), or illicit substances (Chung et al., 2010; Currie & Tough, 2021; Racine et al., 2020; Testa et al., 2022) while pregnant. The most common substances used during pregnancy by U.S. women were (in order of prevalence): tobacco, alcohol, marijuana, opioids (e.g., heroin or misuse of prescription opioids such as methadone, oxycodone, and buprenorphine), misuse of prescription pain relievers (e.g., nonopioids and prescription opioids), misuse of prescription stimulants (e.g., amphetamines), misuse of prescription tranquilizers (e.g., benzodiazepines), cocaine, and methamphetamines (SAMHSA, 2019).

In-utero substance exposure can cause neonatal abstinence syndrome (NAS) (Raffaeli et al., 2017), which may require prolonged hospitalization of the newborn and can have long-term negative consequences on a child’s emotional, behavioral, and general medical health (Anderson et al., 2019; Batty et al., 2006; Gunn et al., 2016; Hudziak & Novins, 2013; Navarrete et al., 2020; Racine et al., 2020; Shonkoff et al., 2012). Substance use in pregnancy not only poses health concerns for offspring in utero and at birth (Anderson et al., 2019; Gunn et al., 2016; Navarrete et al., 2020; Racine et al., 2020), but also has longer-term risks including cognitive (Batty et al., 2006; Navarrete et al., 2020) and behavioral issues (Navarrete et al., 2020). What is particularly concerning is the transgenerational nature of ACEs; that is, those who have experienced ACEs are more likely to engage in behaviors that create similar adversity for their offspring (Knight et al., 2014). For example, infants born to mothers with a substance use disorder (SUD) register an ACEs score of 1 at birth, as substance use by a parent is among the ACEs listed within the household challenges category.

### Implications of SUD in Pregnancy

Prenatal substance exposure is a national health concern, in large part because SUD has reached epidemic proportions in the U.S. Nearly 1 in 15 individuals aged 12 and older (20.4 million in 2019) are living with SUD (Corredor-Waldron & Currie, 2022). In 2021, 49,194 infants across 49 states were born with prenatal substance exposure, up nearly 15% from the 42,821 neonates exposed in 2020 (U.S. Department of Health & Human Services, 2023). Additionally, between 2006 and 2012, the rate of neonatal hospital stays related to substance use increased by 71 percent (from 5.1 to 8.7 per 1000 neonatal stays), and associated aggregate hospital costs increased by 135%, from $253 million to $595 million (Fingar et al., 2015).

The federal child abuse prevention and treatment act (CAPTA) has been amended several times to expand protection of infants affected by maternal substance use during pregnancy. Health care providers are mandated reporters who must alert state agencies when caring for children under age 1 who show signs of substance withdrawal or exposure. The Comprehensive Addiction and Recovery Act of 2016 requires that these infants have a “plan of safe care” in place to ensure the safety and welfare of the infants and provide SUD treatment resources for families after discharge from the care of health providers. In 2018, the substance use-disorder prevention that promotes opioid recovery and treatment (SUPPORT) for Patients and Communities Act further amended CAPTA to earmark federal funding to support the creation of local multidisciplinary teams to develop these plans of safe care (Child Welfare Information Gateway, 2019).

### Local Context for Study

CAPTA prompted a 2018 amendment to Pennsylvania’s Child Protective Services Law (Act 54, Domestic Relations Code, 23 PA.C.S., 2018), requiring health care professionals to alert the state Department of Human Services via form CY-47 when they encounter a newborn showing symptoms of substance exposure. Exposure includes a positive toxicology screen of mother or baby; newborn symptoms of withdrawal, NAS, or fetal alcohol spectrum disorder; or a reported history of maternal substance use during pregnancy—even when it is a prescribed treatment (e.g., maintenance buprenorphine) for SUD. The filing of form CY-47 under Act 54 “shall not constitute a child abuse report,” according to the legislation, but rather ensures that the state can confirm that a plan of safe care is in place for the infant.

**Fig. 1:**
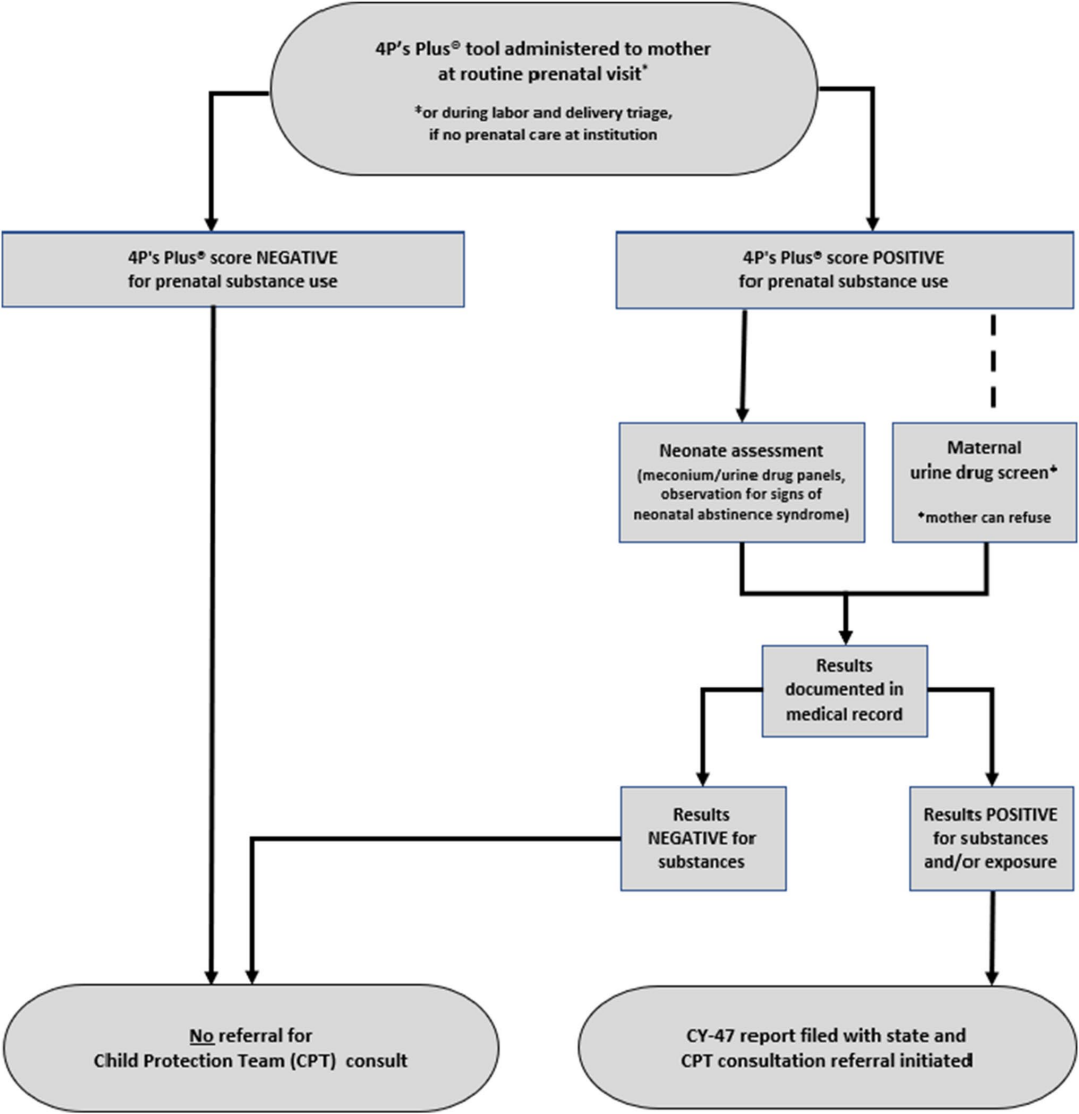
Flowchart to determine initiation of child protection team (CPT) consult

This study takes place at a children’s hospital in southeastern Pennsylvania with an established multidisciplinary team onsite to offer services when concerns about child welfare arise. Filing a CY-47 initiates a consultation with this child protection team (CPT), which includes an interview with the affected infant’s mother, administration of an ACEs questionnaire, and review of toxicology screening results for mother and baby (Fig. 1).

The primary objective of this study was to investigate the prevalence of ACEs in women whose infants were referred to the hospital’s CPT for suspected substance exposure. Researchers hypothesized there would be a higher prevalence of women in this sample with ≥ 4 ACEs than the 15.2% found in the original CDC-Kaiser ACEs study (CDC, 2016; Felitti et al., 1998). The secondary objective was to explore the link between maternal ACEs and substance use during pregnancy, including the specific substances used.

## Methods

### Setting and Population

The children’s hospital at the center of this study is part of a health network that includes primary obstetrical and gynecological services. Routine prenatal care at this network includes administration of the 4P’s Plus® tool, a validated instrument designed to detect maternal alcohol, tobacco, and substance use as well as the individual’s risk for depression or exposure to intimate partner violence (Chasnoff et al., 2007). Results may include use of or exposure to any of the following substances: alcohol, amphetamines, barbiturates, benzodiazepines, cannabinoids, codeine, cocaine, fentanyl, heroin, hydromorphone, inhalants, LSD, methadone, methamphetamine, morphine, MDMA/Ecstasy, phencyclidine, opiates, oxycodone and tobacco. If a pregnant patient who did not receive prenatal care at the institution presents to the hospital’s labor and delivery unit, the 4P’s Plus® screening tool is administered upon admission.

**Fig. 2:**
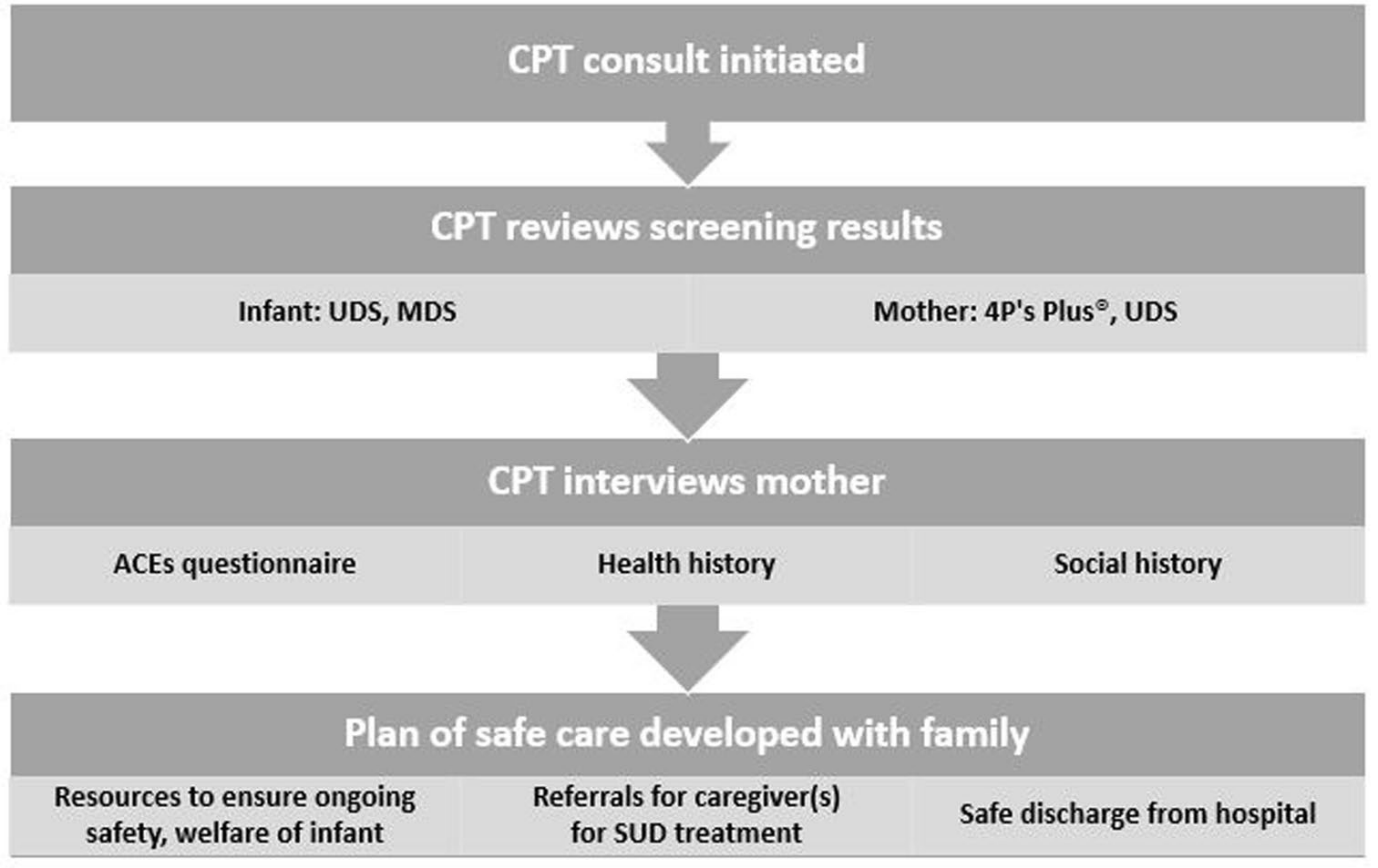
Child protection team screening protocol

If the 4P’s Plus® results indicate substance use during pregnancy, both the mother and the neonate undergo toxicology testing once the neonate is born. Substance use testing for women includes a urine drug screening (UDS) and every positive result is confirmed with secondary laboratory testing to minimize the risk of false positives. In addition, specific urine toxicology screenings are done for fentanyl and buprenorphine. Newborns symptomatic for withdrawal or NAS undergo the same urine screens as their mothers, as well as a meconium drug screen (MDS). Positive results on any of these screenings require the filing of a CY-47 and initiates a consultation with the CPT (Fig. 2). The mother may opt out of toxicology screening, but if a concern for neonatal exposure exists, the infant will be screened as per Pennsylvania’s Act 54. Consent for the toxicology screening is obtained by the attending physician.

### Data Collection

For this retrospective cohort study, researchers reviewed deidentified charts of infants referred to the CPT between September 7, 2017, and November 30, 2020. The study start date coincides with the addition of the ACEs questionnaire to the CPT’s templated consultation note. Inclusion criteria for the study sample was presence of completed maternal ACEs questionnaire in the medical chart. The ACEs questionnaire utilized has been published elsewhere (Nguyen et al., 2019). It was derived directly from the instrument used in the landmark ACEs study (Felitti et al., 1998), which has been shown to have reliable test–retest performance (Dube et al., 2004). The questionnaire asks respondents whether they have experienced any of 10 types of traumatic events before age 18 (Table 1). “Yes” responses are summed to determine the individual’s ACEs score.

**Table 1:** ACEs questionnaire items, adapted from Nguyen et al., 2019

ACE category	Description/questions for mothers
Emotional abuse	Did a parent or other adult in the household often or very often… Swear at you, insult you, put you down, or humiliate you? or Act in a way that made you afraid that you might be physically hurt?
Physical abuse	Did a parent or other adult in the household often or very often… Push, grab, slap, or throw something at you? Ever hit you so hard that you had marks or were injured?
Sexual abuse	Did an adult or person at least 5 years older than you ever… Touch or fondle you or have you touch their body in a sexual way? or Attempt or actually have oral, anal, or vaginal intercourse with you?
Emotional neglect	Did you often or very often feel that… No one in your family loved you or thought you were important or special? or Your family didn’t look out for each other, feel close to each other, or support each other?
Physical neglect	Did you often or very often feel that… You didn’t have enough to eat, had to wear dirty clothes, and had no one to protect you? or Your parents were too drunk or high to take care of you or take you to the doctor if you needed it?
Parental separation/ divorce	Were your parents ever separated or divorced?
Intimate partner violence	Was your mother or mother figure… Often or very often pushed, grabbed, slapped, or had something thrown at her? or Sometimes, often, or very often kicked, bitten, hit with a fist, or hit with something hard? or Ever repeatedly hit over at least a few minutes or threatened with a gun or knife?
Household substance abuse	Did you live with anyone who was a problem drinker or alcoholic, or who used street drugs?
Mental illness in household	Was a household member depressed or mentally ill, or did a household member attempt suicide?
Incarceration in household	Did a household member go to prison?

*ACEs* adverse childhood experiences

Other data points extracted for analysis—but not required for study inclusion—were maternal demographics (age, race, insurance, employment and marital status), maternal UDS results, maternal history of substance use, newborn demographics (age, sex, race), withdrawal presence, MDS results and discharge disposition. This study relied upon MDS rather than UDS results to determine newborn exposure since MDS has greater sensitivity for detecting substances in newborns (Ostrea et al., 1989). Maternal substance use (including any history of substance use and substance use during pregnancy) came from any information available in the chart, including the UDS or specific urine toxicology results, the 4P’s Plus® tool, and/or notes from the CPT consultation interview.

Study data were collected and managed using research electronica data capture (REDCap), a secure, web-based software platform designed to support data collection for research studies (Harris et al., 2009). The health network’s institutional review board granted ethical approval for this study.

### Statistical Methods

Descriptive statistics captured newborn and maternal demographics, toxicology results, and substance use during pregnancy. Frequencies and percentages were reported for categorical variables, while means/standard deviations or medians/interquartile ranges were reported as appropriate for continuous variables. ACEs questionnaire results were represented as both a cumulative score (0–10) and as a categorical variable using a cut-point (4), based upon previous literature demonstrating that women who experienced ≥ 4 ACEs had the highest risk of adverse outcomes (Chung et al., 2010; Currie & Tough, 2021; Frankenberger et al., 2015; Leeners et al., 2014; Nguyen et al., 2019; Olsen, 2018; Racine et al., 2020; Testa et al., 2022). ACEs categories (abuse, neglect, and household challenges) also were analyzed and created based upon whether a woman had any of the individual ACEs contained within that category (e.g., woman is positive for the abuse category if she experienced emotional, physical, or sexual abuse before age 18).

The hypothesis for this research is based on the published research demonstrating that women experiencing ≥ 4 ACEs have increased odds of substance use in pregnancy. To assess the primary objective (comparing the proportion of women in this study with ≥ 4 ACEs with the 15.2% found in the original CDC-Kaiser ACEs study), the Chi-square goodness of fit test was used. To determine whether associations existed between ACEs (i.e., individual ACEs, ACE categories, dichotomized using a cut-point of 4 and total ACEs score) and substance use during pregnancy, bivariate analyses were performed. The Chi-square test of independence or Fisher’s exact test was utilized for categorical variables and the independent samples t-test was used for continuous variables. Furthermore, adjusted binomial logistic regression models were used to explore the relationship between ACEs (using a cut-point of 4) and substance use during pregnancy (yes/no), controlling for demographic characteristics. Individual models were constructed for the most prevalent substances used (methamphetamine, cannabinoid, heroin, tobacco) and any substance use during pregnancy as outcomes.

Substance use during pregnancy was defined as confirmation (by self-report or urine screen) of at least one substance used during the gestational period. Each model used the ACEs score cut-point (< 4/ ≥ 4) as the primary exposure, the reference group for ACEs score were mothers with < 4 ACEs, therefore substance use was compared between mothers with ≥ 4 ACEs to those with < 4 ACEs and controlled for maternal age, marital status and employment status. Education level was unavailable for most women in this sample and race could not be included due to vast underrepresentation, yielding unreliable estimates. Model covariates were determined a priori, based upon review of the literature (Chung et al., 2010; Nguyen et al., 2019). Model assumptions—including linearity, multicollinearity, and the presence of influential outliers—were assessed and met. The Hosmer–Lemeshow goodness of fit χ^2^ statistic was calculated for each model to assess model fit. Alpha was set to 0.05 for all analyses. Statistical analysis was done using SAS 9.4 software [Copyright© (2016) SAS Institute Inc. SAS and all other SAS Institute Inc. product or service names are registered trademarks or trademarks of SAS Institute Inc., Cary, NC, USA.]

## Results

During the 3 year study period, the CPT performed 235 consults. Of those, 222 newborn charts met inclusion criteria. There were four sets of twins in this dataset; therefore, analyses included data from 218 mothers.

### Newborn Demographics and Exposure

The median age of newborns upon consult was three days (IQR 1–4), with 53.2% male. The majority with an identified race (n = 212) were white/Caucasian (67.9%). Half the infants (50.0%) had withdrawal symptoms or NAS, and 64.0% of those infants were treated pharmacologically. MDS results most frequently detected cannabinoids (32.4%), followed by buprenorphine (29.3%), and methadone (28.8%). Of the 47 neonates positive for amphetamine exposure, most (93.6%) were confirmed positive for methamphetamines. There were no newborn deaths in this cohort. The majority (86.5%) were discharged home with their mothers. Demographic characteristics and toxicology results for the newborns are presented in Table 2.

**Table 2:** Newborn characteristics and substance exposure (*n* = 222)

Age, median (IQR), days	3.0 (1.0–4.0)
Sex	
Male, *n* (%)	118 (53.2)
Race/ethnicity (*n* = 212), *n* (%)	
White or Caucasian	144 (67.9)
Hispanic or Latino	58 (27.4)
Black or African American	6 (2.8)
Asian or Pacific Islander	2 (0.9)
Presence of withdrawal, *n* (%)	
Yes	111 (50.0)
Pharmacologically treated for withdrawal or NAS (*n* = 111), *n* (%)	
Yes	71 (64.0)
MDS positive results, *n* (%)	
Amphetamines	47 (21.2)
If + for Amphetamines, did it include methamphetamine? (*n* = 47) (Yes)	44 (93.6)
Barbiturates	5 (2.3)
Benzodiazepines	6 (2.7)
Buprenorphine	65 (29.3)
Cannabinoids	72 (32.4)
Cocaine	20 (9.0)
Methadone	64 (28.8)
Opiates	45 (20.3)
Phencyclidine	0
Negative/None	16 (7.2)
Unavailable	3 (1.4)
Disposition at discharge, *n* (%)	
Home	192 (86.5)
Foster care	27 (12.2)
Transfer	3 (1.4)
Death	0

I*QR* interquartile range, *NAS* neonatal abstinence syndrome, *MDS* meconium drug screening

### Maternal Demographics and Substance Use

The mean maternal age was 29.2 years (SD 5.2), with the majority (89.0%) of mothers on Medicaid. Among those with charted demographic information (n = 211, Table 3), most were white/Caucasian (80.1%) and married or in a relationship (74.4%). Of those with noted employment status (n = 217), most identified as unemployed/homemaking (66.4%).

**Table 3:** Maternal characteristics and substance exposure (*n* = 218)

Age, mean (SD)*,* years	29.2 (5.2)
Medicaid, n (%)	194 (89.0)
Race/ethnicity, *n* (%), (*n* = 211)	
White/Caucasian	169 (80.1)
Hispanic/Latino	31 (14.7)
Black/African American	9 (4.3)
Asian/Pacific Islander	2 (0.9)
Employment status, *n* (%), (*n* = 217)	
Unemployed/homemaker	144 (66.4)
Employed	73 (33.6)
Marital status, *n* (%), (*n* = 211)	
Married/in relationship	157 (74.4)
Single/separated	54 (25.6)
UDS results, *n* (%)	
Positive	146 (67.0)
Negative/none	70 (32.1)
Unavailable/declined	2 (0.92)
Reported substance use in pregnancy, *n* (%)	
Positive	185 (84.9)
Treated for SUD during pregnancy, *n* (%)	
Yes	122 (56.0)
History of substance misuse, *n* (%)	
Yes	206 (94.5)

*SD* standard deviation, *SUD* substance use disorder

**Fig. 3:**
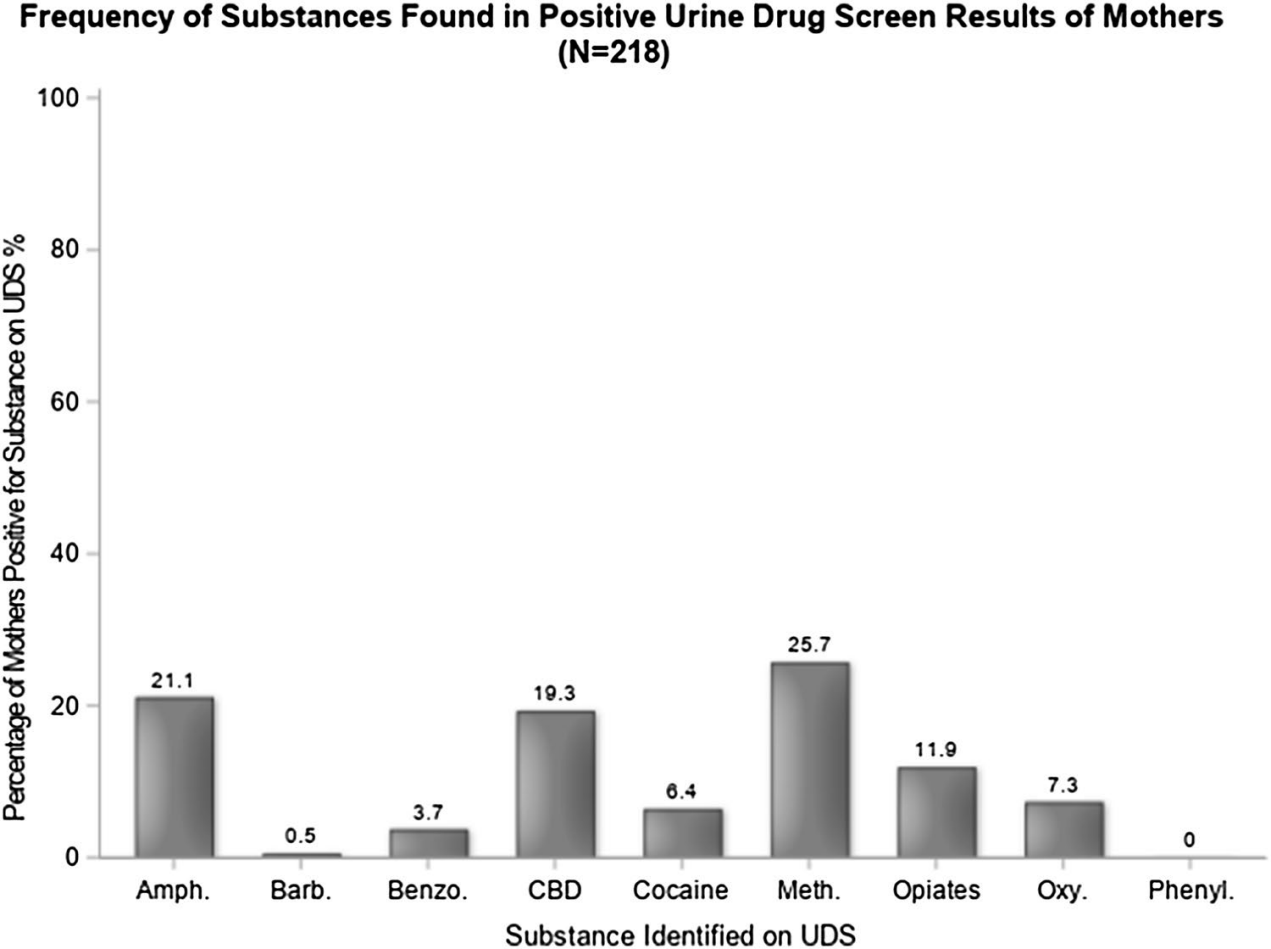
Frequency of substances found in positive maternal UDS (*n* = 218). UDS urine drug screens, Amph amphetamines, Barb barbiturates, Benzo benzodiazepines, CBD cannabinoids, Meth methadone. Oxy oxycodone, Phenyl phencyclidine

**Fig. 4:**
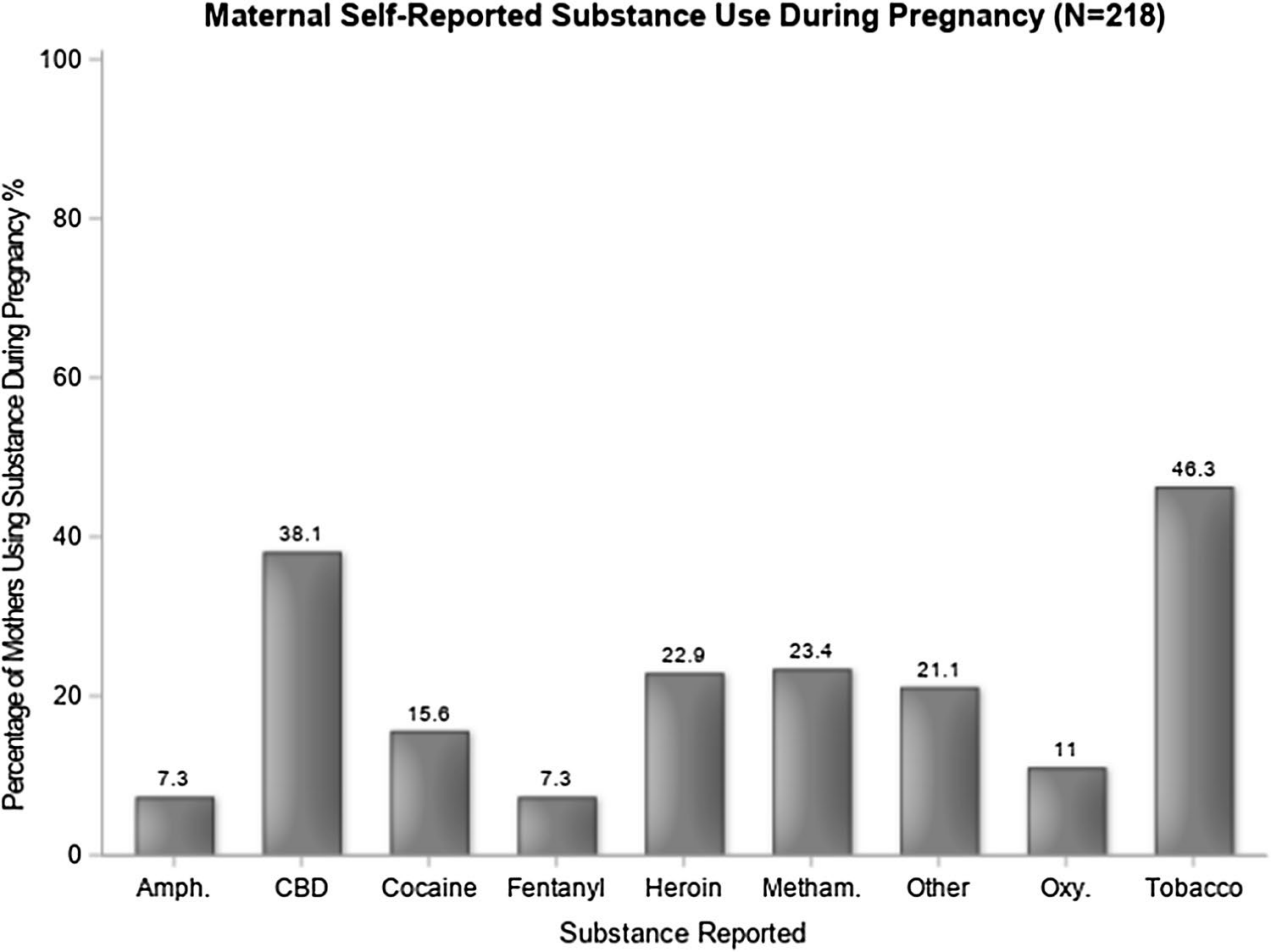
Frequency of self-reported substance use during pregnancy (*n* = 218). Other” category includes alcohol, benzodiazepines, codeine, hydrocodone, methadone, morphine, 3,4-Methylenedioxymethamphetamine (MDMA, ecstasy), and phencyclidine. Amph amphetamines, CBD cannabinoids, Meth methadone. Oxy oxycodone

All mothers included in the study (n = 218) consented to toxicology tests upon CPT consult. A majority (67.0%) yielded positive results (Fig. 3), and 84.9% of mothers self-reported substance use during pregnancy (Fig. 4). A large majority of women (94.5%) had a history of substance misuse, and more than half (56.0%) had received treatment for SUD while pregnant.

### Maternal ACEs

The results of the maternal ACEs questionnaire are displayed in Table 4. The mean total ACEs score (range 0–10) was 3.6 (SD 2.6), with 50.9% (95%CI 44.1%, 57.7%) of women experiencing ≥ 4 ACEs. The individual ACE types are grouped by category (abuse, neglect, household challenges) and stratified by maternal reporting of substance use during pregnancy (yes/no).

**Table 4:** Distribution of maternal ACEs in the entire sample and stratified by substance use During pregnancy (*n* = 218)

ACEs category		Substance use during pregnancy?		*P* value
	Total *n* = 218 *n* (%)	No *n* = 33 *n* (%)	Yes *n* = 185 *n* (%)	
Abuse (Any)	97 (44.5)	12 (36.4)	85 (46.0)	0.31
Emotional	59 (27.1)	6 (18.2)	53 (28.7)	0.21
Physical	48 (22.0)	5 (15.2)	43 (23.2)	0.30
Sexual	70 (32.1)	9 (27.3)	61 (33.0)	0.52
Neglect (Any)	69 (31.2)	6 (18.2)	62 (33.5)	0.08
Emotional	61 (28.0)	5 (15.2)	56 (30.3)	0.07
Physical	28 (12.8)	4 (12.1)	24 (13.0)	1.00
Household challenges (any)	187 (85.8)	24 (72.7)	163 (88.1)	0.03
Parental separation/divorce	155 (71.1)	15 (45.5)	140 (75.7)	< 0.001*
Intimate partner violence	69 (31.7)	9 (27.3)	60 (32.4)	0.56
Household substance abuse	117 (53.7)	16 (48.5)	101 (54.6)	0.52
Mental illness in household	98 (45.0)	12 (36.4)	86 (46.5)	0.28
Incarceration in household	77 (35.3)	14 (42.4)	63 (34.1)	0.35
ACEs score (cut-point 4)				0.50
< 4 ACEs	107 (49.1)	18 (54.6)	89 (48.1)	
≥ 4 ACEs	111 (50.9)	15 (45.5)	96 (51.9)	
Total ACEs score** mean (SD)	3.6 (2.6)	2.9 (2.5)	3.7 (2.6)	0.09
Total ACEs score** range	0–10	0–9	0–10	–

*ACE* adverse childhood experience, *SD* standard deviation

*Statistically significant, **Score range: 1–10

Household challenges (85.8%) represented the most prevalent ACEs category experienced by women in this cohort, followed by abuse (44.5%), and neglect (31.2%). The most frequently reported individual ACEs type was living in a household where parental separation or divorce occurred (71.1%), followed by substance use in the household (53.7%), and mental illness in the household (45.0%).

### ACEs and Substance Use in Pregnancy

Stratifying by reported substance use during pregnancy (yes/no) revealed that for each ACEs category, a greater proportion of women who responded “yes” to substance use also had experienced ACEs in that category (household challenges: 88.1% yes vs. 72.7% no; abuse: 46.0% vs. 36.4%; neglect: 33.5% vs. 18.2%). Experiencing a household challenges ACE (p = 0.03), and specifically being in a household where separation or divorce occurred (p < 0.001) were significantly associated with substance use during pregnancy. No other significant associations were found between individual ACE types or ACEs categories and substance use during pregnancy. However, the same pattern emerged for individual ACEs as with the ACEs categories: More women reporting specific types of ACEs also noted substance use in pregnancy. This was true for all individual ACEs except having a household member go to prison. This pattern remains true when stratifying ACEs scores using the cut-point of four (51.9% with ≥ 4 ACEs vs. 48.1% with < 4 ACEs had used substances during pregnancy).

### Risks Associated with Maternal Substances of Choice

**Fig. 5:**
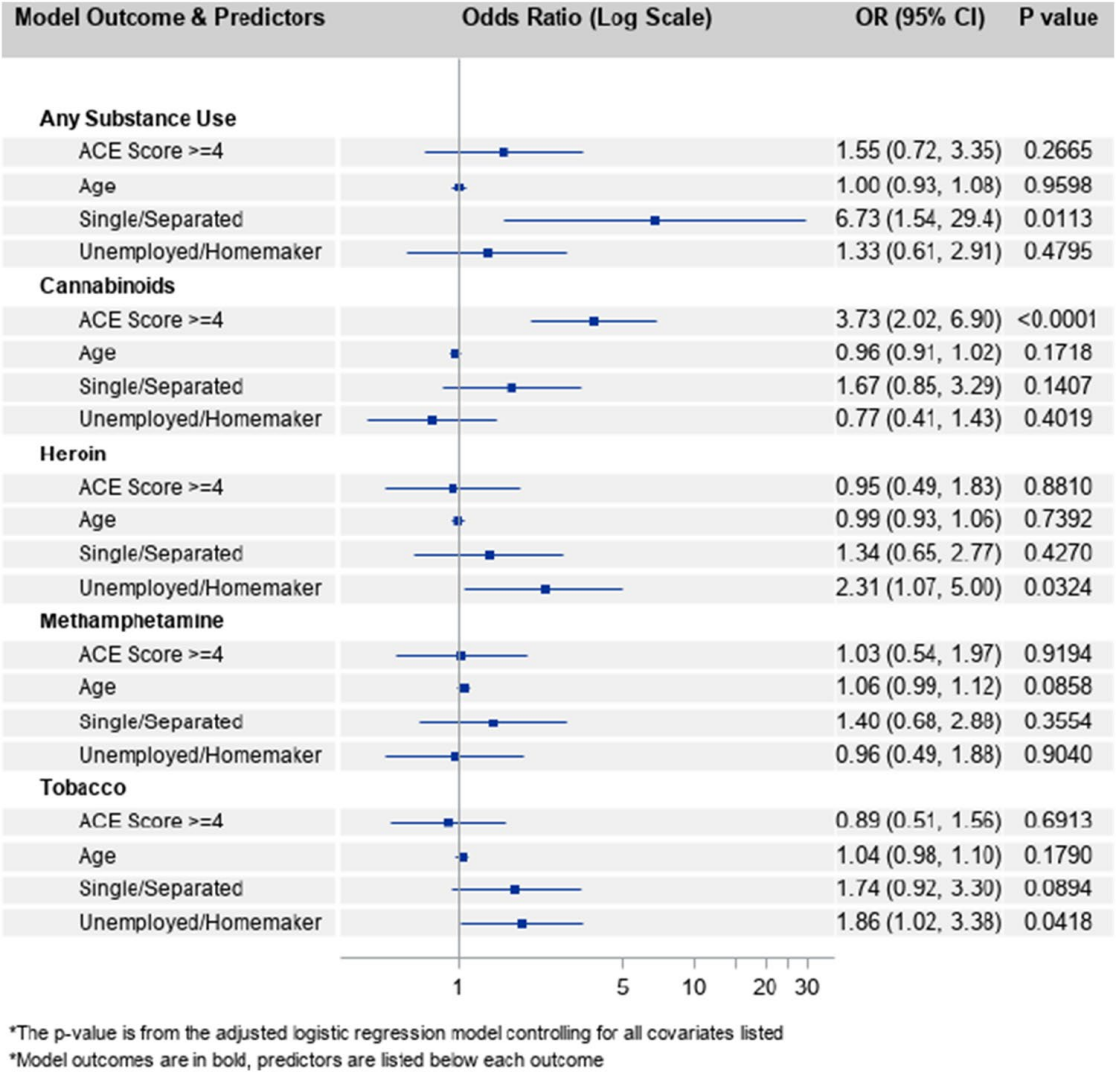
Adjusted odds ratios (log scale) from binomial logistic regression models predicting use of any substance, cannabinoids, heroin, methamphetamine, or tobacco during pregnancy (*n* = 218). ACE adverse childhood experience, OR odds ratio, CI confidence interval

The multivariate binomial logistic regression models (Fig. 5) for the four most prevalent substances used by women in this sample revealed additional risk factors. The use of cannabinoids during pregnancy was significantly higher (aOR 3.73, 95%CI 2.02, 6.90, p < 0.001) among women with ≥ 4 ACEs compared with women with < 4 ACEs, controlling for age, relationship, and employment status. Cumulative ACEs score was not a significant predictor in any of the other models. However, women who were unemployed or homemakers were about twice as likely to use heroin (aOR 2.31, 95%CI: 1.07, 5.00; p = 0.03) or tobacco products (aOR 1.86, 95%CI 1.02, 3.38; p = 0.04) during pregnancy compared with women who were employed, controlling for all other variables in the model. Single or separated women were significantly more likely (aOR 6.73, 95%CI 1.54, 29.43; p = 0.01) to report use of any substance during pregnancy compared with those who were married or in a relationship.

## Discussion

As hypothesized, this study of pregnant people referred for CPT consultations contained a higher prevalence of those with ≥ 4 ACEs scores than found in the original CDC-Kaiser ACEs study (50.9% vs. 15.2%). The prevalence in the current study also exceeded that seen in Merrick et al.’s expanded ACEs study (2018), which found ≥ 4 ACEs among 17.8% of U.S. women. Nguyen et al. (2019) also saw a lower prevalence of ≥ 4 ACEs within a diverse population of pregnant people in outpatient (22.0%) and inpatient (16.0%) settings.

The statistically significant association found between prenatal substance use and ACEs in the household challenges category—in particular, parental separation or divorce—mirrors the findings of previous studies. Currie and Tough (2021) found that pregnant people who engaged in binge drinking, substance use, and smoking had a higher prevalence of ACEs in the household challenges category, while Racine et al. (2020) saw a medium effect size in their analysis of whether these types of ACEs predicted substance use in pregnancy. Analysis of other types of ACEs, such as childhood sexual abuse, have shown mixed results in previous studies (Currie & Tough, 2021; Leeners et al., 2014; Racine et al., 2020). In the current study, other types of ACEs did not reach statistical significance when analyzed for associations with substance use in pregnancy.

### Maternal Substance Use

Previously published research has shown a dose–response relationship between increased ACEs scores and use of tobacco, alcohol and illicit substances, not only in the general population (Scheidell et al., 2018), but also among pregnant people (Chung et al., 2010; Currie & Tough, 2021; Frankenberger et al., 2015; Racine et al., 2020; Testa et al., 2022). This study found that only cannabinoid use in pregnancy had a significant association with those who reported ≥ 4 ACEs.

However, the prevalence of prenatal substance use found in this study (84.9% reported, 67.0% confirmed with screening) is much higher than the 3% to 23%, depending on substance type, found in other studies (Chung et al., 2010; Currie & Tough, 2021; Racine et al., 2020). It is not surprising that cannabinoids were the most frequently detected substances in both infants and their mothers, given that cannabis is the most consumed substance in the U.S. (Azofeifa et al., 2016; Navarrete et al., 2020) as well as the substance most frequently utilized by pregnant people (Ko et al., 2015; SAMHSA, 2019). However, the prevalence of cannabinoid use in this study was far greater than the estimated 7% to 8% of women and 4% to 5% of pregnant people who have reported using cannabis in the U.S. (Brown et al., 2017; Ko et al., 2015; SAMHSA, 2019). Medicalization and legalization have normalized usage of cannabis nationwide, and some worry that the changing national landscape contributes to misinformation about its safety and efficacy (Navarrete et al., 2020; Sarvet et al., 2018). Pennsylvania law still limits marijuana use to those for whom it is deemed medically necessary.

### Limitations and Opportunities for Future Research

This study has several limitations. Because the population of interest was narrowly defined as neonates referred for consultation with the CPT, there was no control group. This likely contributed to the high prevalence of those in the sample who screened positive for substance use and ACEs. Maternal education, race, and ethnicity also were underrepresented in this sample. Any self-reporting of childhood adversity is subject to recall bias, and the same can be said for any of the self-report tools used (e.g., 4P’s Plus®, history of substance use). Also, because all CPT consult charts since the 2017 addition of the ACEs questionnaire were reviewed for analysis, no a priori sample size calculation was conducted. Therefore, the effect size of these results was undetermined.

Prevalence of both maternal substance use and newborn exposure may be underreported in this sample. Universal toxicology testing for pregnant people and their infants does not occur in this health network. If infants were exposed to substances in utero and did not exhibit symptoms of withdrawal prior to discharge or if they were exposed to substances that do not create symptoms (such as marijuana or methamphetamine), their exposure may have been missed. In some cases, infants with a negative UDS are discharged home before the slower-to-process MDS returns as positive, and thus CPT was not consulted. To assess the true prevalence of in-utero substance exposure and maternal ACEs, future studies would ideally include a larger sample size with a control group. Comparison of neonatal outcomes by type of substance used and impact of polysubstance use would offer more insight on this topic.

### Implications for Practice

Between 2017 and 2020, overdose deaths among pregnant and postpartum people increased 81% (Bruzelius & Martins, 2022). This study offers further evidence that SUD among pregnant people is a concern. Buprenorphine and methadone were the second- and third-most frequently detected substances in newborn screens, and methadone was reported by one-quarter of the women interviewed by the CPT.

It is important to note that the legal landscapes for perinatal substance use vary widely between U.S. states. This study occurred in a state where substance use in pregnancy is not treated as child abuse and where mandated reporting exists to ensure that a plan of safe care is in place to support the health and welfare of the infant and family, including connecting caregivers with needed SUD treatment resources. It has been shown that pregnant people residing in states with punitive policies for substance use during pregnancy are less likely to obtain timely or quality prenatal and postpartum care (Austin et al., 2022) and have higher rates of infants born with neonatal abstinence syndrome (Faherty et al., 2019). In addition, stigmatization of SUD in pregnant people is still pervasive among clinicians (Kelly & Westerhoff, 2010). Implementing selective screening, therefore, may re-enforce this stigma.

SUD is a mental health diagnosis and should not be criminalized. The American Medical Association, American College of Gynecologists (ACOG), National Perinatal Association, American Academy of Pediatricians (AAP), American Academy of Family Physicians and American Society of Addiction Medicine are just a few of the professional societies that have spoken out against incarceration or other criminal penalties for substance use in pregnancy (Pregnancy Justice, 2021). These organizations cite concerns such as reduced fetal health due to avoidance of prenatal care by mothers fearing punishment, breakdown of clinician-patient trust that interferes with optimal care delivery, and failure to diagnose and treat women with SUD and exposed infants. Another concern is that an infant born exposed to substances already has experienced one ACE. Incarcerating the mother increases the infant’s ACEs score to 2, thus perpetuating the generational impact of ACEs.

Universal screening, therefore, should be conducted with informed consent of the pregnant individual, and positive results must “not be a deterrent to care, a disqualifier for coverage under publicly funded programs, or the sole factor for determining family separation,” as recommended by ACOG (2023) and the AAP (Patrick et al., 2020). Precedent for universal screening of newborns—including for hearing loss, serious inherited disorders, critical congenital heart disease, and HIV—has demonstrated that early identification and intervention can improve outcomes. Identifying and supporting pregnant women with high ACEs scores and/or SUD may further support treatment engagement and improved outcomes for these women and their infants. In addition to connecting women to SUD treatment resources, clinicians can offer women to trauma-focused therapy or provide health education counseling. Setting up home nurse visits after discharge would provide ongoing support for mother-infant dyads. The American Academy of Pediatrics recommends that infants exposed to opioids be seen within 48 h of discharge and again 1 week after that (Patrick et al., 2020).

## Conclusion

This study adds further evidence that women with multiple ACEs are at higher risk for prenatal substance use, thus illustrating the intergenerational impact of childhood trauma. Embedding an ACEs screening tool into routine prenatal care offers opportunities for early intervention to support women at risk of poor outcomes of ACEs, such as SUD. Universal, nonpunitive toxicology screening for both pregnant people and their infants increases the likelihood that all impacted individuals would be detected and could obtain the intervention services they need. Supporting women with SUD, rather than penalizing them, should be a public health priority.

## Data Availability

Not applicable.
